# Discovery and Validation of a Six-Marker Serum Protein Signature for the Diagnosis of Active Pulmonary Tuberculosis

**DOI:** 10.1128/JCM.00467-17

**Published:** 2017-09-25

**Authors:** Mary A. De Groote, David G. Sterling, Thomas Hraha, Theresa M. Russell, Louis S. Green, Kirsten Wall, Stephan Kraemer, Rachel Ostroff, Nebojsa Janjic, Urs A. Ochsner

**Affiliations:** SomaLogic, Inc., Boulder, Colorado, USA; Carter BloodCare & Baylor University Medical Center

**Keywords:** aptamer, biomarker, proteomics, tuberculosis

## Abstract

New non-sputum biomarker tests for active tuberculosis (TB) diagnostics are of the highest priority for global TB control. We performed in-depth proteomic analysis using the 4,000-plex SOMAscan assay on 1,470 serum samples from seven countries where TB is endemic. All samples were from patients with symptoms and signs suggestive of active pulmonary TB that were systematically confirmed or ruled out for TB by culture and clinical follow-up. HIV coinfection was present in 34% of samples, and 25% were sputum smear negative. Serum protein biomarkers were identified by stability selection using L1-regularized logistic regression and by Kolmogorov-Smirnov (KS) statistics. A naive Bayes classifier using six host response markers (HR6 model), including SYWC, kallistatin, complement C9, gelsolin, testican-2, and aldolase C, performed well in a training set (area under the sensitivity-specificity curve [AUC] of 0.94) and in a blinded verification set (AUC of 0.92) to distinguish TB and non-TB samples. Differential expression was also highly significant (*P* < 10^−20^) for previously described TB markers, such as IP-10, LBP, FCG3B, and TSP4, and for many novel proteins not previously associated with TB. Proteins with the largest median fold changes were SAA (serum amyloid protein A), NPS-PLA2 (secreted phospholipase A2), and CA6 (carbonic anhydrase 6). Target product profiles (TPPs) for a non-sputum biomarker test to diagnose active TB for treatment initiation (TPP#1) and for a community-based triage or referral test (TPP#2) have been published by the WHO. With 90% sensitivity and 80% specificity, the HR6 model fell short of TPP#1 but reached TPP#2 performance criteria. In conclusion, we identified and validated a six-marker signature for active TB that warrants diagnostic development on a patient-near platform.

## INTRODUCTION

Tuberculosis (TB) remains a major global health problem, causing the highest mortality of any single infectious disease worldwide in 2015, and is among the top 10 causes of all deaths worldwide. The latest reports suggest that while substantial progress has been made in reducing TB incidence, there are still two in five individuals who remain undiagnosed and contribute to the spread of the disease. The United Nations global strategy for TB control has the goal of a 90% reduction in TB deaths and an 80% reduction in TB incident rates by 2030 (1). To achieve these goals, new diagnostic tools are critically important and vital for controlling the TB epidemic (2, 3). Better tests using non-sputum samples, such as blood, will broaden access to diagnostics that can inform treatment and halt the spread of disease in communities (4). No current diagnostic test is accurate and cheap enough, and existing tests often are inadequate in children and those with extrapulmonary disease (2–4). Empirical treatment is still commonplace, especially when the suspicion of TB or the risk of mortality from untreated TB is high (5).

Transformative technology like GeneXpert MTB/RIF has already begun to improve outcomes but only when combined with operational and infrastructure improvements (6). Despite the worldwide rollout of GeneXpert tests, there are still significant unmet diagnostic needs. Most notably, reliable non-sputum-based tests that could be performed at low cost at district levels and health posts are critically needed (7, 8). A sensitive triage test could be used to identify those who need confirmatory molecular or culture-based testing, including drug susceptibility testing (DST), at a higher level of the health system. A high-specificity (≥98%) test for TB would allow treatment to be initiated following a positive result but would need to retain sensitivity sufficiently high (>65%) to be useful in countries where TB is highly endemic.

Multiple “omics” studies have discovered biomarkers of active TB (9–11), including transcriptomic approaches using RNA sequencing (12, 13) and microRNA detection (14), proteomic studies via mass spectrometry to detect peptides after proteolytic cleavage of proteins (15, 16), immune-based studies typically using T cell responses (17), and metabolomics using mass spectroscopy (18–20). Mass spectroscopy is limited by cost and sensitivity concerns, can be labor-intensive, and has a relatively low throughput for biomarker discovery (21). Mass spectroscopy is also technically more challenging as a patient-near application compared to simple assay formats such as lateral flow or other sandwich-type assays using antibodies or aptamers for quantitation of specific markers. Biomarker studies published to date are often difficult to compare, since they focus on one specific geographic area, vary in the numbers of subjects, and often enroll control populations without reported information on latent TB status or specifics on other infectious or inflammatory diseases. Since 2003, the Foundation for Innovative New Diagnostics (FIND; Geneva, Switzerland) has curated a high-quality worldwide specimen bank to catalyze academics and industry toward better TB diagnostic tests. A large number of serum samples were graciously made available for our work using the SOMAscan assay. This proteomic platform measures >4,000 proteins simultaneously in a small volume (50 μl) of plasma or serum, has a dynamic range of ∼8 logs, a median lower limit of detection of 40 fM, and high precision (<5% coefficient of variation). Unbiased approaches to biomarker discovery using SOMAscan have led to diagnostic blood protein signatures for a variety of diseases affecting the lung, including TB (22–24), non-small-cell lung cancer (25), and mesothelioma (26).

Using SOMAscan, we embarked on a multiple-phase study to determine whether protein abundances from the human host can be sufficiently robust to meet the challenges in TB diagnostics. It is very difficult to detect pathogen-derived markers directly in blood with meaningful sensitivity (27) without employing sophisticated methodologies, such as mass spectrometry, to enhance the detection of Mycobacterium tuberculosis-specific peptides in digested serum samples (28).

The studies we performed were designed to assess the ability of serum protein biomarkers to distinguish TB-positive subjects (confirmed by positive M. tuberculosis sputum culture) from non-TB subjects presenting with TB-like symptoms in the presence and absence of HIV. The results presented here demonstrate that robust signals from host protein biomarkers are able to reproducibly distinguish TB from non-TB subjects. Additionally, suites of markers were identified which correlated with early treatment response and which could be useful for detection and monitoring of drug-resistant TB (29–31).

## RESULTS

### Study subjects and TB biomarker discovery.

Toward the goal of improving upon our previous phase I biomarker study (see Fig. S1 in the supplemental material) that had produced a 9-protein model with 80% sensitivity and 84% specificity (Fig. S2), we used a larger version of the SOMAscan and a geographically more diverse discovery sample set. The serum samples had been collected from patients at multiple clinics in seven countries: South Africa, Peru, Zimbabwe, Uganda, Vietnam, Colombia, and Bangladesh (Table 1). For biomarker discovery, a total of 252 non-TB and 252 TB samples were tested on SOMAscan, using the version that measured 4,156 analytes. A small fraction of the samples (7.5%) were removed because they were hemolyzed (*n* = 7), failed the assay of quality control metrics (*n* = 15), or were duplicates (*n* = 16). The remaining 466 samples were deemed fit for data analysis and included 159 (TB negative, HIV negative), 151 (TB positive, HIV negative, including 36 smear negative), 79 (TB negative, HIV positive), and 77 (TB positive, HIV positive, including 23 smear negative) samples. There were inherent demographic differences: TB patients were younger (*P* = 0.0050), had a lower body mass index (BMI) (*P* = 0.0012), and had a higher proportion of males (*P* = 0.0010) than the non-TB group. The sample classes were, by design, well balanced with respect to HIV status (Fig. S3).

**TABLE 1:** Demographic and clinical data for participants in TB serum biomarker studies using the 4,000-plex (phase II) SOMAscan assay

Demographic or clinical parameter	Phase II sample set (tested on 4,000-plex)	
	Training	Blinded verification
No. of samples tested	504	216
South Africa	198	83
Peru	84	22
Vietnam	90	30
Bangladesh	50	40
Zimbabwe	18	15
Uganda	24	0
Colombia	40	26
Active TB, culture confirmed [no. (%)]	252 (50)	93 (43)
Smear-positive TB, no.	189 (75% of cases)	71 (76% of cases)
Smear-negative TB, no.	63 (25% of cases)	22 (24% of cases)
Included in analysis [no. (%)]	466 (92.5)	204 (94.4)
HIV coinfection [no. (%)]	170 (34)	67 (31)
Age, yr (range), no.^*a*^	34 (17–76), 446	34 (18–79), 199
Gender (% male), no.^*a*^	61.5, 423	63.5, 159
Weight, kg (range), no.^*a*^	60 (40–129), 137	60 (40–121), 57
Height, cm (range), no.^*a*^	164 (140–189), 155	165 (146–188), 70
BMI, kg m^−2^ (range), no.^*a*^	21.6 (15.3–45.3), 124	20.4 (14.6–34.6), 46

a Reported for a subset of the samples only, as indicated by the number of samples.

**FIG 1:**
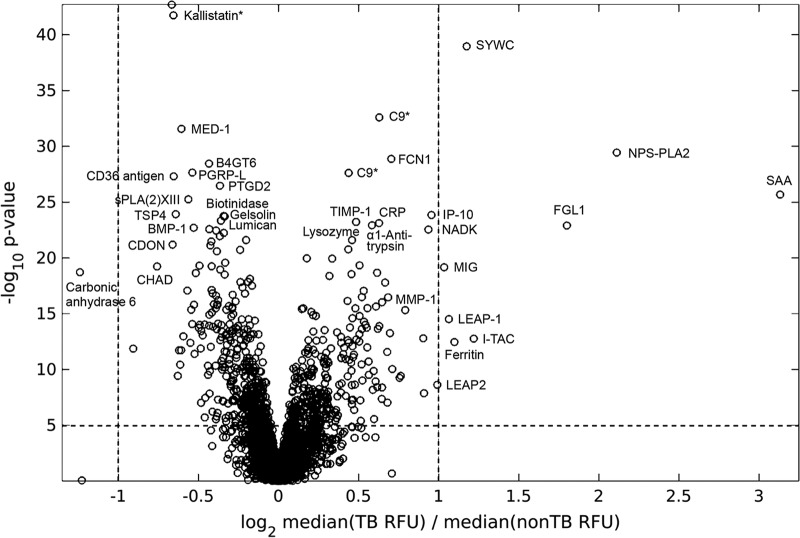
Volcano plot of the differential expression of proteins measured in serum samples from TB (*n* = 228) compared to non-TB (*n* = 238) subjects. The statistically most significant markers are shown toward the top, and proteins with the largest fold change of the median signals are toward the right and left, respectively. Kallistatin and C9 are shown twice, because two separate SOMAmers for each of these proteins were used in SOMAscan, as indicated by an asterisk.

At a 5% Bonferroni-corrected significance level, the Kolmogorov-Smirnov (KS) test identified 722/4,156 (17%) proteins differentially expressed between TB and non-TB groups. Of these, 312/722 (43%) were expressed at higher levels in TB patients than in non-TB patients. The major significantly differentiating proteins are depicted in the volcano plot (Fig. 1). In addition to this univariate biomarker discovery analysis, we applied stability selection using an L_1_-regularized logistic regression model. This method allowed the inclusion of clinical and demographic metadata along with all protein measurements when comparing TB and non-TB samples and typically yields the most robust and noncorrelated markers (Fig. S4).

The top markers distinguishing TB from non-TB samples are summarized in Table 2. Kallistatin, SYWC, and C9 were the top three markers identified in both analyses. We confirmed the markers previously found in phase I, including those with large KS distances (kallistatin, C9, NPS-PLA2, IP10, TSP4, and gelsolin) and those with large median fold changes (SAA, CRP, and carbonic anhydrase 6). Many new markers were discovered among the larger proteomic content of the 4,000-plex SOMAscan version compared to the 1,129-plex scan used in phase I, including SYWC, MED-1, FCN1, B4GT6, PGRP-L, CD36 antigen, PTGD2, sPLA(2)-XIII, biotinidase, lumican, α1-antitrypsin, FGL1, BMP-1, CD109, cathelicidin, and aldolase C.

**TABLE 2:** Serum protein markers that distinguish TB from non-TB among initial TB suspects, ranked by KS statistic^*a*^

Target	SwissProt accession no.	Signal comparison TB vs non-TB			Stability selection^*b*^	
		Signed KS^*b*^	*P* value	Fold change^*c*^	Selection probability	Stability curve area
Kallistatin	P29622	−0.644	2.00 × 10^−43^	1.58 ↓	0.785	0.162
SYWC	P23381	0.616	1.12 × 10^−39^	2.36 ↑	1	0.28
C9	P02748	0.564	2.54 × 10^−33^	1.54 ↑	0.885	0.154
MED-1	Q15648	−0.555	2.77 × 10^−32^	1.47 ↓		
NPS-PLA2	P14555	0.536	3.71 × 10^−30^	4.05 ↑		
FCN1	O00602	0.531	1.36 × 10^−29^	1.52 ↑	0.585	0.09
B4GT6	Q9UBX8	−0.527	3.56 × 10^−29^	1.33 ↓		
PGRP-L	Q96PD5	−0.519	2.30 × 10^−28^	1.41 ↓		
CD36 ANTIGEN	P16671	−0.516	4.99 × 10^−28^	1.53 ↓	0.588	0.093
PTGD2	O60760	−0.508	3.43 × 10^−27^	1.29 ↓	0.665	0.095
SAA	P0DJI8	0.501	2.15 × 10^−26^	7.99 ↑		
sPLA(2)-XIII	Q9BX93	−0.497	5.64 × 10^−26^	1.42 ↓		
TSP4	P35443	−0.483	1.22 × 10^−24^	1.53 ↓		
IP-10	P02778	0.483	1.44 × 10^−24^	1.95 ↑		
Biotinidase	P43251	−0.482	1.78 × 10^−24^	1.25 ↓		
Gelsolin	P06396	−0.481	1.93 × 10^−24^	1.26 ↓		
Lumican	P51884	−0.478	4.69 × 10^−24^	1.29 ↓		
TIMP-1	P01033	0.476	6.03 × 10^−24^	1.38 ↑		
CRP	P02741	0.475	7.62 × 10^−24^	1.51 ↑		
α1-antitrypsin	P01009	0.473	1.21 × 10^−23^	1.48 ↑		
FGL1	Q08830	0.473	1.26 × 10^−23^	3.48 ↑		
BMP-1	P13497	−0.471	1.98 × 10^−23^	1.41 ↓	0.512	0.059
CD109	Q6YHK3	−0.470	2.63 × 10^−23^	1.35 ↓		
NADK	O95544	0.470	2.85 × 10^−23^	1.74 ↑		
COMP	P49747	−0.469	3.51 × 10^−23^	1.33 ↓		
MRC2	Q9UBG0	−0.463	1.14 × 10^−22^	1.27 ↓		
Lysozyme	P61626	0.459	2.18 × 10^−22^	1.34 ↑	0.517	0.046
CDON	Q4KMG0	−0.456	6.38 × 10^−22^	1.57 ↓		
suPAR	Q03405	0.442	1.10 × 10^−20^	1.12 ↑		
C1QT1	Q9BXJ1	0.442	1.16 × 10^−20^	1.23 ↑		
LRIG3	Q6UXM1	−0.438	2.68 × 10^−20^	1.25 ↓		
Nr-CAM	Q92823	−0.434	5.74 × 10^−20^	1.30 ↓		
CHAD	O15335	−0.434	5.38 × 10^−20^	1.65 ↓		
MIG	Q07325	0. 433	6.94 × 10^−20^	2.13 ↑		
Carbonic anhydrase 6	P23280	−0.428	1.92 × 10^−19^	2.32 ↓		
HSP 90a/b	P07900, P08238	0.428	2.15 × 10^−19^	1.47 ↑		
FCRL1	Q96LA6	−0.409	8.51 × 10^−18^	1.45 ↓	0.757	0.077
Apo F	Q13790	0.402	3.50 × 10^−17^	1.52 ↑		
Aldolase C	P09972	−0.395	1.46 × 10^−16^	1.28 ↓	0.555	0.033
MMP-2	P08253	−0.394	1.51 × 10^−16^	1.28 ↓		
Cathepsin V	O60911	−0.394	1.57 × 10^−16^	1.39 ↓		
MMP-1	P03956	0.388	4.84 × 10^−16^	1.61 ↑		
IL-6	P05231	0.383	1.32 × 10^−15^	1.17 ↑		
LEAP-1	P81172	0.378	3.05 × 10^−15^	1.85 ↑		
Cathelicidin peptide	P49913	0.360	6.88 × 10^−14^	1.37 ↑	0.598	0.04
I-TAC	O14625	0.355	1.73 × 10^−13^	2.46 ↑		
Testican-2	Q92563	−0.354	2.04 × 10^−13^	1.17 ↓	0.933	0.077
Ferritin	P02794, P02792	0.351	3.45 × 10^−13^	2.01 ↑		
P5I13	Q8NBR0	0.344	1.06 × 10^−12^	1.43 ↑	0.603	0.044
CK-MB	P12277, P06732	−0.341	1.93 × 10^−12^	1.56↓		
Macrophage mannose receptor	P22897	0.332	7.88 × 10^−12^	1.38 ↑		
CNDP1	Q96KN2	−0.321	4.56 × 10^−11^	1.24 ↓	0.713	0.041
LEAP2	Q969E1	0.294	2.36 × 10^−09^	1.90 ↑		
LBP^*d*^	P18428	0.552	7.33 × 10^−29^	1.90 ↑		
DERM^*d*^	Q07507	−0.537	2.88 × 10^−27^	1.43 ↓	0.598	0.05
ITI heavy chain H4^*d*^	Q14624	0.528	2.14 × 10^−26^	1.43 ↑		
Afamin^*d*^	P43652	−0.526	3.32 × 10^−26^	1.64 ↓	0.813	0.16
BGH3^*d*^	Q15582	−0.502	6.55 × 10^−24^	1.49 ↓	0.703	0.11
FCG3B^*d*^	O75015	0.425	3.05 × 10^−17^	1.40 ↑	0.81	0.06

a Shown are markers with a KS distance of >0.4, markers identified by stability selection, and markers that showed a median fold change of at least 1.25-fold between TB and non-TB samples. Signed KS statistics indicate the direction of the differential expression (positive values indicate higher in TB than non-TB samples).

b Phase II training samples (*n* = 466) were used for stability selection and KS statistics.

c All phase II unique samples (*n* = 618) were used to calculate the median fold change (upward arrows indicate higher median signals in TB than non-TB samples).

d Data shown for phase I training samples (*n* = 333). These markers were among the top 50 in phase I but not in phase II due to technical and analytical issues.

A new top marker more abundant in TB than non-TB samples was SYWC, a gamma interferon (IFN-γ)-inducible Trp-tRNA-synthetase associated with stress response. Target identity of the HR6 proteins was confirmed by affinity capture (pulldown) from serum, using bead-immobilized slow off-rate modified aptamers (SOMAmers) as described in another study (27). The limited sensitivity of this method allowed pulldown of only medium- to high-abundance serum proteins, including kallistatin, C9, gelsolin, and SYWC (Fig. S5).

### Modeling and validation of a host marker signature for active TB in adults.

**FIG 2:**
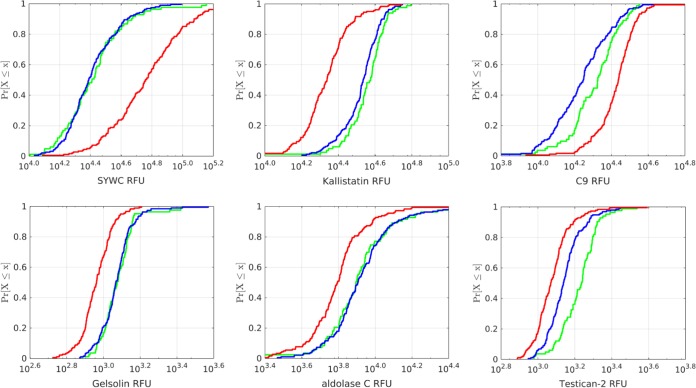
Cumulative distribution function (CDF) plots of the six host markers comprising the HR6 model (SYWC, kallistatin, complement C9, gelsolin, aldolase C, and testican-2) in a training set of 181 TB (red) versus 190 non-TB (blue) samples. Also shown are measurements in samples from Spain and Canada (green) obtained from patients with lung disorders other than TB.

The identification of additional strong serum protein markers for TB prompted an effort to build an improved model with increased sensitivity and specificity and possibly fewer proteins compared to the HR9 model (Fig. S2). The samples were randomly split into a training set containing 80% of the samples (*n* = 190 non-TB versus *n* = 181 TB) and a test set containing the remaining 20% of samples (*n* = 48 non-TB versus *n* = 47 TB) that were used for independent evaluation of preliminary model performance. Only the training set was used to construct and establish the models. Candidate TB markers that were associated with demographic variables or by clinical parameters other than TB status were dismissed. Examples were CCL28, which was a good TB marker in samples from South Africa but not from other sites, and sCD163, which distinguished TB from non-TB only in the HIV-positive subpopulation (Fig. S6). In addition, we required candidate markers to have good correlation of measurements between serum and plasma, so subsequent models could be applied to either type of sample matrix. Lastly, candidate TB biomarkers were deprioritized if they showed extremely narrow signal distributions which made them less robust with respect to the standard hybridization and median normalization procedures typically applied to SOMAscan data. Using these criteria, base models were constructed, followed by either backward elimination or forward selection using a subset of the markers shown in Table 2. Comparison of cross-validated candidate model performance with the goal to maximize either area under the concentration-time curve (AUC), specificity, or sensitivity and specificity resulted in a final naive Bayes model using Gaussian class-specific densities with robust parameter estimates. This optimal model contained 6 host response proteins (HR6 model): SYWC, kallistatin, C9, gelsolin, testican-2, and aldolase C. Four of the six biomarkers had been part of the previous HR9 model from phase I, and SYWC and aldolase C were discovered in the larger, 4,000-plex SOMAscan used in phase II and provided additional sensitivity and specificity to the model. In the training set, the HR6 model showed an AUC of 0.93 (95% confidence interval [95% CI], 0.90, 0.95), which was slightly better than the performance of the previous HR9 model refit to the new data (AUC of 0.89 [95% CI, 0.85, 0.92]). Model performance was confirmed in the 20% holdout test set of 95 samples that had not been used for building the model (AUC of 0.88 [95% CI, 0.79, 0.94]). Importantly, the model performance was not significantly different when stratified by country, gender, or age (data not shown). The HR6 model was also applied to a specificity challenge set that included 82 samples (40 from Canada and 42 from Spain) from non-TB patients with pulmonary disease such as chronic obstructive pulmonary disease, bronchiectasis, asthma, pneumonia, bronchitis, dyspnea, talc-induced lung disease, or emphysema. These samples were from an older population (median age, 64.5 years; range, 19 to 93 years) and included 22 subjects with a history of TB and 9 patients with confirmed nontuberculous mycobacterial (NTM) infection. HR6 performed quite well in this challenge set (93% specificity), with 6 false positives, including one gross misclassification (log odds of >5) and only one of the nine NTM patients misclassified as TB (log odds of 1.67), indicating that the specificity of the HR6 model was not affected by the presence of NTM. Similarly, the HR6 model performed well in a control set of 168 samples from a healthy normal population for which we had SOMAscan data on file (>98% specificity), with 3 weak false positives. The separation of TB and non-TB samples is shown by cumulative distribution function (CDF) plots for each of the HR6 markers shown in Fig. 2.

**FIG 3:**
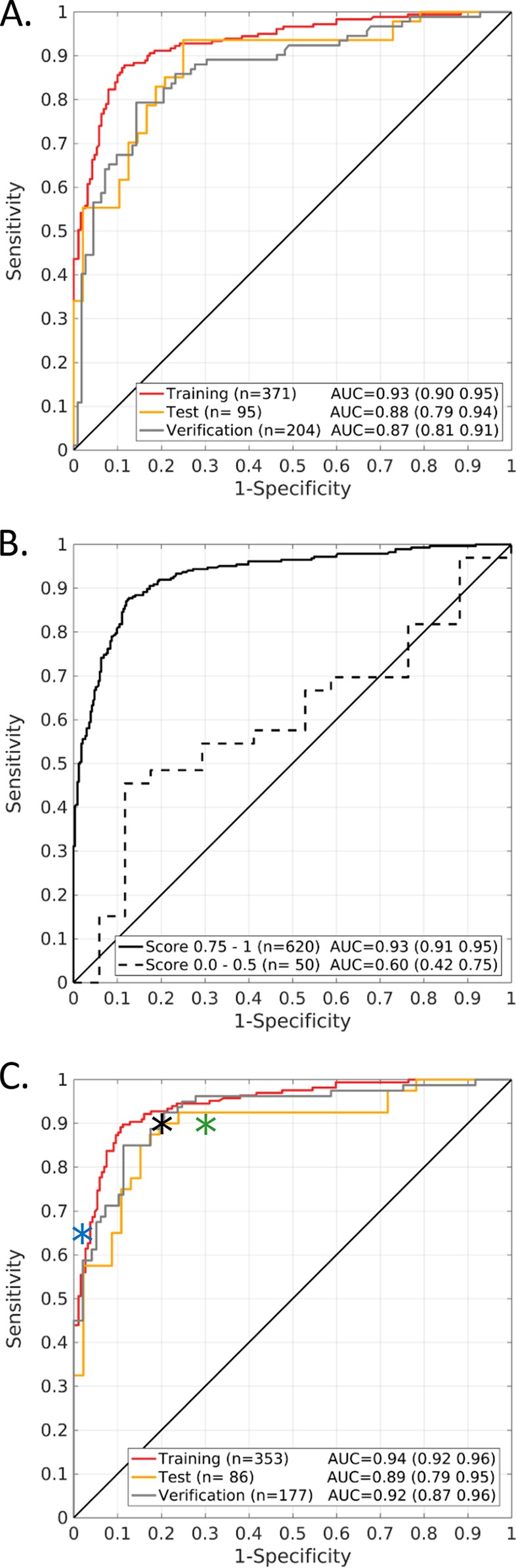
ROC curves for the initial assessment of HR6 model performance to distinguish TB from non-TB serum samples. (A) All phase II samples (*n* = 670), stratified by training, test, and blinded verification set. (B) Stratification by the sample metadata confidence score. The vast majority of samples (*n* = 620; 92.5%) were associated with strong metadata that provided a high level of confidence in their true classification, while a small subset of samples (*n* = 50; 7.5%) had less convincing metadata. (C) HR6 model performance in phase II samples with high-confidence metadata, stratified by training, test, and blinded verification set. The optimal operative point for the HR6 ROC curve was at 90% sensitivity and 80% specificity, as indicated by the black asterisk. For reference, the minimal performance requirements for TPP#1 (test to diagnose active TB) and for TPP#2 (triage/referral test) are shown as blue and green asterisks, respectively.

The HR6 model was locked down and shared with FIND prior to testing a blinded verification set of 216 samples, again including some replicate samples (*n* = 19) to enable bridging of the separate data sets. Predictions using the HR6 model were made for 212 samples, excluding three hemolyzed samples and one assay failure, and the calculated log odds for TB were submitted to FIND. Unblinding of the metadata revealed eight samples from subjects with incomplete clinical data, which were removed from the analysis. In the 204 remaining verification samples the HR6 model performance showed an AUC of 0.87 (95% CI, 0.81, 0.91), which was well within the expected sensitivity and specificity at the Bayes operating points based on bootstrap estimates of the associated empirical 95% confidence intervals. Receiver operating characteristic (ROC) curves for the 670 samples stratified by training, test, and blinded verification sets are shown in Fig. 3A.

### Misclassifications, data discrepancies, and sample quality scores.

The HR6 model produced several gross misclassifications in training, test, and verification sets, prompting further analysis of the proteomic data and the associated metadata. The clerical error rate in data collection, labeling, aliquoting, and database management appeared to be low for the 670 samples analyzed in phase II. Signals for gender-specific proteins (FSH, LH, and PSA) were generally very consistent with the metadata. One subject had discrepant entries for age and gender compared to metadata for a separate serum aliquot from the same subject provided 2 years apart. Another subject was undoubtedly pregnant based on the proteomic profile but was annotated as male. Among a total of 64 subjects for whom duplicate aliquots had been received and tested separately during phase I and II assay runs, 58 showed highly concordant proteomic measurements (rho of >0.98) typical for replicates from the same subject. The other 6 paired samples, however, were clearly discordant (rho of <0.90), as typically seen for unrelated individuals, and four of these were samples that had been relabeled for blinding purposes for use in a verification set. We also reexamined the quality and quantity of metadata provided with the samples and devised a score to determine how confident we were about the true diagnosis of TB (scores: 1, perfect; 0.75, good; 0.5, incomplete data; 0.25, questionable; 0, no data), as shown in detail in Fig. S7A. In essence, a good metadata confidence score required consistent culture data on at least two sputa for TB and complete follow-up information for the non-TB subjects. Overall, the sample metadata were very good, with 95% of the non-TB and 89% of the TB samples having a score of ≥0.75. Samples with poor metadata confidence scores were more frequent among the HR6 misclassifications, and consequently the HR6 model performed much better in high-confidence samples (AUC of 0.93 [95% CI, 0.91, 0.95]) than in the low-confidence samples (AUC of 0.60 [95% CI, 0.42, 0.75]), as shown in ROC curves (Fig. 3B). Eliminating the 50 samples with questionable metadata and dismissing the 4 discordant duplicates resulted in a modest increase of the model performance in the remaining 616 samples (Fig. 3C). In the blinded verification set (*n* = 177), the AUC increased from 0.87 (95% CI, 0.81, 0.91) to 0.92 (95% CI, 0.87, 0.96) in the high-confidence samples. Cross-tabulation of the HR6 test results for the 616 samples by the FIND reference standard indicated that there was no statistical difference in HR6 model performance between the training and the blinded verification sets (Table 3). The log odds distributions for these 616 samples illustrating the misclassifications are depicted in Fig. S7B. The HR6 model performance was independently calculated and verified by FIND personnel.

**TABLE 3:** Cross-tabulation of HR6 SOMAscan test results by the reference test for all phase II training, test, and blinded verification samples with complete metadata^*a*^

SOMAscan (HR6) group	Reference status (FIND)	HR6 result (no.)		Sensitivity (%)	Specificity (%)	PPV (%)	NPV (%)	Accuracy (%)
		Pos.	Neg.					
All (*n* = 616)	Pos.	252	34	88.1	87	85.4	89.4	87.5
	Neg.	43	287					
Training (*n* = 353)	Pos.	149	17	89.8	87.7	86.6	90.6	88.7
	Neg.	23	164					
Test (*n* = 86)	Pos.	35	5	87.5	80.4	79.5	88.1	83.7
	Neg.	9	37					
Verification (*n* = 177)	Pos.	68	12	85	88.7	86.1	87.8	87
	Neg.	11	86					

a The cutoff for separation of TB from non-TB was log odds of >0. Also shown are sensitivity, specificity, positive predictive value (PPV), negative predictive value (NPV), and accuracy. Pos., positive; Neg., negative.

### Effect of HIV coinfection and smear status on HR6 model performance.

Duplicate samples (*n* = 47) that were part of both discovery and blinded verification were removed, and the remaining set of unique, high-confidence samples (*n* = 569) was used for a more detailed calculation of the sensitivity and specificity of the HR6 model in different subject subgroups. ROC curves to distinguish TB (*n* = 262) from non-TB subjects (*n* = 307) stratified by HIV status indicated only slightly reduced test accuracy for HIV-positive TB subjects (*n* = 85; AUC of 0.93 [95% CI, 0.87, 0.96]) compared to HIV-negative TB subjects (*n* = 177; AUC of 0.94 [95% CI, 0.92, 0.96]) but a sharper drop in smear-negative TB subjects (*n* = 52; AUC of 0.87 [95% CI, 0.79, 0.92]) compared to smear-positive TB subjects (*n* = 210; AUC of 0.95 [95% CI, 0.93, 0.97]) (Fig. S7C). Among the 569 unique samples there were 21 gross misclassifications, including 10 false negatives (log odds of <−5), 6 of which were from subjects with smear-negative TB. Sensitivity and specificity of HR6 for active pulmonary TB detection in all unique samples (*n* = 569) are summarized in Table 4, stratified by HIV coinfection and smear status. To be even more stringent, we determined the model performance after eliminating all training samples, thereby limiting the analysis to the combined test and verification samples only (*n* = 216). ROC curves to distinguish TB (*n* = 96) from non-TB subjects (*n* = 120) were again stratified by HIV infection and smear status (Fig. S7D). HR6 performance dropped in HIV-positive TB subjects (*n* = 30; AUC of 0.88 [95% CI, 0.75, 0.95]) compared to HIV-negative TB subjects (*n* = 66; AUC of 0.94 [95% CI, 0.89, 0.97]) and in smear-negative TB (*n* = 11; AUC of 0.77 [95% CI, 0.53, 0.91]) compared to smear-positive TB subjects (*n* = 85; AUC of 0.94 [95% CI, 0.89, 0.97]).

**TABLE 4:** Sensitivity and specificity of HR6 for active pulmonary TB detection, stratified by HIV coinfection and smear status

PTB global cohort (*n* = 569) HIV status	HR6 positive (no. positive/total no.)	% sensitivity (95% CI)	% specificity (95% CI)
HIV negative (*n* = 403)			
Culture-positive TB	156/177	88.1 (80.8–92.1)	
Smear positive	132/142	93.0 (85.2–95.0)	
Smear negative	24/35	68.6 (62.9–80.0)	
Non-TB	24/226		89.4 (85.8–90.1)
HIV positive (*n* = 166)			
Culture-positive TB	76/85	89.4 (82.3–91.8)	
Smear positive	62/68	91.2 (85.3–92.6)	
Smear negative	14/17	82.4 (70.6–88.2)	
Non-TB	14/81		82.7 (72.8–87.7)

### Design of a targeted TB panel assay and alternative models.

**FIG 4:**
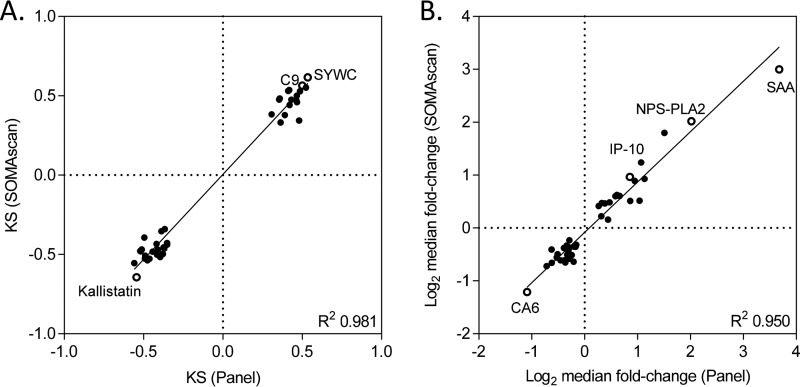
Concordance of a focused TB panel with the full 4,000-plex SOMAscan. (A) KS distances for 40 relevant markers based on full SOMAscan assay of training samples compared to a targeted TB panel assay of a subset of TB (*n* = 39) and non-TB (*n* = 35) samples. The top three markers based on KS statistics (kallistatin, SYWC, and C9) are shown as open circles. (B) Median fold change for the same 40 markers, based on measurements on full SOMAscan data of all phase II unique samples and in the panel assay of subsets of TB (*n* = 39) and non-TB (*n* = 35) samples, respectively. Markers with large median fold changes (SAA, NPS-PLA2, IP-10, and CA6) are shown as open circles.

A targeted panel contained a small subset of the entire SOMAscan menu, including the SOMAmer probes for the TB markers shown in Table 2. This allowed the readout on a much smaller, low-density slide array (Applied Microarrays, Inc.), resulting in a more economical assay format. The panel assay was performed in semiautomated as well as manual format with a representative subset of TB (*n* = 39) and non-TB (*n* = 35) samples. The TB biomarkers discovered on full SOMAscan were confirmed on the focused panel with regard to both KS statistics and the fold change of the median between TB and non-TB samples (Fig. 4).

HR6 contained an optimal number of robust markers to reach the desired performance in a training set (AUC of 0.93). For comparison, much simpler models with only the two statistically most significant markers (kallistatin and SYWC) or with the four proteins showing the largest fold changes between TB and non-TB samples (SAA, NPS-PLA2, IP-10, and CA6) had clearly inferior performance in the same set (Fig. S8).

## DISCUSSION

We conducted a large, multiphase study to identify and quantify serum protein markers indicative of active pulmonary TB disease. The SOMAscan assay provided a sensitive, high-throughput platform to generate proteomic measurements for over 4,000 proteins in nearly 1,500 serum samples quickly and accurately. By following standard bioinformatics approaches, the most significant and robust biomarkers were identified and subsequently used to build a small proteomic model (HR6) which was then successfully verified in blinded verification samples.

Adding strength to our results is that we found many previously discovered proteins (23), and the proteomic data are biologically and clinically plausible. The biologic categories of the markers encompass immunity and host defense (C9, FCG3B, cathelicidin, LBP, and FCRL1), vascular remodeling (kallistatin and TSP4), extracellular matrix and tissue remodeling (gelsolin, COMP, lumican, testican-2, and CD36 antigen), lipid transport and regulation (phospholipases), coagulation and complement (C1QT1), proteases (MMP-1, MMP-2, CNDP1, and BMP-1), and bacterial recognition (PGRP-L). Among the top markers is kallistatin, and we have previously reported that kallistatin levels increased following an 8-week intensive phase of TB therapy (23), which is consistent with our findings here of lower kallistatin levels in TB compared to non-TB serum. Kallistatin, a kallikrein protease inhibitor, has antiangiogenic, antioxidant, antiapoptotic, and anti-inflammatory properties, and its concentration in blood is decreased in cases of bacterial pneumonia (32). SYWC is an interesting protein not previously described as a putative diagnostic marker and was found to be highly discriminatory in serum of active human TB. SYWC is the interferon gamma-inducible, cytoplasmic form of tryptophanyl-tRNA synthetase (also called WARS or TrpRS). Tryptophan is essential for M. tuberculosis (33), particularly for the synthesis of a coat protein important for survival in the macrophage (34). Upregulation of SYWC in the host leads to restriction of free tryptophan, and tryptophan depletion is lethal to M. tuberculosis (35). SYWC has very recently been found upregulated in human THP-1 cells infected with M. tuberculosis (36). In a separate mechanism during TB infection, tryptophan often becomes limiting due to its conversion to kynurenine by indoleamine 2,3-dioxygenase (37). This enzyme was in fact among the top 5% of markers in our SOMAscan analysis and was indeed upregulated in TB (KS of 0.33), although not as strongly as SYWC. The finding of non-cytokine markers such as SYWC expands the biology of TB proteomics that may be less prone to variation by other concomitant diseases.

Our biomarker data are in good overall agreement with a study by Achkar et al. using serum collected at New York hospitals, where markers for active TB were identified via liquid chromatography and mass spectrometry (15). Our markers and signatures performed well in HIV-infected subjects despite the fact that HIV greatly alters the lung milieu (38). Roughly one-third of our study population was HIV positive, and biomarker discovery and model building were performed in the full sample set, although we did deprioritize TB markers that were affected by HIV status. In contrast, the study by Achkar (15) reported separate lists of serum markers and signatures for HIV-negative and HIV-positive groups.

TB has evolved with humans for thousands of years, and our broad proteomic approach to discover interesting biological functions may yield important insights into how this pathogen came to be so successful (39). More detailed protein-protein interactions with regard to the serum markers described in this study can be seen using the String database function shown in Fig. S9 in the supplemental material. Interestingly, several of the pulmonary TB markers have previously been reported as part of a 7-protein classifier for lung cancer, including C9, CRP, and carbonic anhydrase (25), and C9 and kallistatin were part of a 13-marker signature of mesothelioma (26). The overlap is not surprising given the similarities with respect to the chronic nature of these lung diseases, involving common host defense pathways of immune responses and tissue repair and remodeling. However, our additional markers add specificity for M. tuberculosis infection to the HR6 model.

The next goal is to develop a rapid, inexpensive, and practical platform that can be rolled out in the appropriate settings. Target product profiles (TTPs) for TB diagnostics for non-sputum samples have been prioritized with input from key experts (40) and have subsequently been adopted and published by the WHO (41). The first TPP is for a rapid biomarker-based diagnostic test with very high specificity (98%) that could be used as a standalone or confirmatory test. The second TPP is for a triage or referral test that has high sensitivity (90 to 95%) but modest specificity (70 to 80%) and could be used to identify people with presumptive TB or to rule out TB if negative. As can be seen in the ROC curves presented in Fig. 3C, the HR6 model meets the performance criteria for the referral/triage test but not for a TB detection test. The optimal operating point of the HR6 model was at 90% sensitivity and 80% specificity overall, although test accuracy was reduced in HIV-positive compared to HIV-negative TB patients and was also lower in smear-negative TB than in smear-positive TB. The HR6 misclassification error rates in subjects from high- and middle-burden countries were much higher than diagnostic errors for the subjects from Spain and Canada, where the TB burden is lower, suggesting that our signatures are sensitive to subclinical incipient disease. Latent infection has been increasingly recognized as a spectrum of disease, and it is interesting to speculate that some of the false positives are due to subclinical disease (42). Although we did not train the model in low-TB-burden areas, there were no gross misclassification errors in a large collection of healthy subject samples from the United States.

With regard to reaching the TPPs, particularly for a TB detection test with ≥98% specificity, the accuracy of a “truth standard” is critical to avoid falsely classified samples that will reduce the apparent performance of a new model. Clearly, the HR6 model performed much better in the subset of samples with high metadata confidence scores compared to the subset of samples (7.5%) with low confidence scores.

Our hope is that this test could be developed and rolled out for active case finding in the tier of the health care system that is linked to appropriate case management and treatment capability to maximize impact. Much work has been done in this area by analysis of the effect of GeneXpert, which is paving the way for new and accurate TB diagnostics (6). While the Xpert MTB/RIF assay is a major step forward, it does not work well on serum or blood. The urine LAM test performs well only in HIV-positive subjects with low CD4 cell counts. We hope our test will be more sensitive across the CD4 spectrum. Such a test could be a useful screening tool utilizing non-sputum samples in all patient strata. Moreover, a protein biomarker panel test could increase case detection, shorten diagnostic delay, and reduce transmission when rolled out in the context of a functional health care system (4, 43–46). If transitioned to a cheaper, patient-near platform, it could be rolled out in peripheral health care posts to reach the largest fraction of patients seeking care, but additional domestic or donor funding would be necessary to realize these goals (47).

## MATERIALS AND METHODS

### Study design and sample collection.

FIND and its partner sites in South Africa, Peru, Zimbabwe, Uganda, Vietnam, Colombia, and Bangladesh enrolled adults who presented with signs and symptoms of TB (cough for at least 2 weeks, fevers, weight loss, and night sweats). Basic demographics, such as age, weight, and gender, and clinical metadata, such as HIV status and, in some cases, CD4 cell counts and viral load, were collected. Chest radiographs were performed and interpreted by local radiologists. Sputum samples were obtained for acid-fast staining, solid and liquid cultures, and, occasionally, GeneXpert MTB/RIF testing. Subjects with culture positivity on either or both solid (Lowenstein-Jensen) and liquid (mycobacterial growth indicator tube) media were considered confirmed TB cases. Those with more than 5 days of prior TB chemotherapy were excluded. Criteria for ruling out TB were defined by FIND and included culture and smear negativity and resolution of symptoms in the absence of specific TB therapy at follow-up at 2 to 3 months.

All serum samples were obtained at baseline, given a unique barcode, and frozen on site in 0.5-ml aliquots prior to shipment to a central repository. Ethical approval was obtained by FIND at the sites. Some of the samples were from the original WHO/TDR specimen bank now managed by FIND, including sera from Colombia and Uganda, and a set of non-TB samples from Spain and Canada. A separate sample set of healthy control sera from Covance, Inc., was available from SomaLogic's biorepository and was also analyzed. SomaLogic received only deidentified samples.

Frozen serum aliquots were received at SomaLogic in multiple shipments for the two phases of study (Table 1; see also Fig. S1A in the supplemental material). Phase I and phase II were run approximately 2 years apart and differed in the diversity of geographical origin of the samples and in the number of proteins for which the serum levels were determined. Each phase of the study was designed to have an initial, nonblinded biomarker discovery and model-building stage which included samples from subjects chosen by FIND. The blinded verification stage involved a separate shipment of a sample set chosen by FIND, and no diagnostic classification or clinical metadata were included. The key for sample class description was kept securely at the FIND headquarters and not shared with SomaLogic until proteomic analyses were completed, and diagnostic predictions on the blinded samples were deposited at FIND.

### Proteomic analysis (SOMAscan).

The version of SOMAscan in use at the time when samples arrived was applied, which was the 1,129-plex in phase I and the 4,000-plex in phase II. SOMAscan is a proprietary multiplexed workflow to detect relative abundance of signals representing proteins recognized by slow off-rate modified aptamer (SOMAmer) reagents and has been described in greater detail elsewhere (22). In brief, SOMAmer reagents for over 4,000 proteins had been generated in a procedure known as systematic evolution of ligands by exponential enrichment (SELEX) to bind to their cognate target with high affinity and specificity (48, 49). The DNA libraries contain modified deoxyuridine bases that harbor hydrophobic moieties at the 5 position, which typically results in superior binding properties of SOMAmers compared to standard aptamers (50). During SOMAscan, the SOMAmer reagents form stoichiometric complexes with their cognate targets and are ultimately released and hybridized to an array (Agilent), resulting in relative fluorescence units (RFU) as a readout that is proportional to the concentration of the corresponding protein in the sample. The analytes measured in the 4,000-plex SOMAscan include secreted proteins (47%), extracellular domains (28%), and intracellular proteins (25%) of broad biological groups, such as receptors, kinases, cytokines, proteases, growth factors, protease inhibitors, hormones, and structural proteins.

Target specificity has been confirmed for a subset of analytes via affinity capture (pulldown) assays using bead-immobilized SOMAmers followed by fluorescent tagging of the analytes and SDS-PAGE analysis as described previously (27). This method was applicable only to high- and medium-abundance serum proteins due to its limited sensitivity for low-abundance proteins when using small (<0.5-ml) serum volumes.

For all SOMAscan assays, samples were aliquoted into two-dimensional-barcoded matrix tubes (ThermoScientific), and standard dilutions of serum (0.005%, 1%, and 40%) were made to accommodate the wide concentration range of different serum proteins in normal individuals and to capture a large dynamic range of protein concentrations. A total of 1,470 samples were tested, and 92.5% of them were fit for analysis. Samples were excluded from the analysis if they showed signs of gross hemolysis (apparent reddish color or abnormally high hemoglobin-to-haptoglobin ratio measured by SOMAscan), as was noted for 53 (3.6%) of the samples or if they failed the assay due to technical issues (low sample volume, clogging of filter well, incomplete photocleavage, or hybridization), which would have necessitated excessive median normalization scale factors outside the allowable range of 0.4 to 2.5. Duplicate samples were averaged and included for data analysis unless they had discordant proteomic or demographic data (*n* = 6). Verification samples that were not truly blinded (*n* = 11) or had insufficient clinical data (*n* = 8) were also excluded. Quality control and calibrator samples were run in parallel and included normal healthy local blood donors. Bridging samples were used to allow for calibration of data sets obtained on different testing occasions.

### Statistical analysis and modeling.

The two-tailed *t* test was used to determine statistical differences in the demographic and clinical metadata between TB and non-TB patients. All protein data were log transformed to stabilize the variance. Median normalization was used to adjust for sample-specific assay bias, and the scale factors ranged from 0.4 to 2.5. Nonparametric statistical tests were used for all comparisons: the Kolmogorov-Smirnov (KS) test for univariate hypothesis testing and the Kruskal-Wallis test for intersite comparisons within each diagnostic category. While the KS statistic is an unsigned quantity, we report a signed KS statistic where the plus or minus sign indicates the direction of the differential expression. Benjamini and Hochberg false discovery rates were used to adjust *P* values for multiple comparisons (51). Stability selection using L1-regularized logistic regression was used to identify stable features in the presence of the available clinical covariates (52). The candidate biomarkers were combined using a naive Bayes classifier to create a diagnostic model that generates the probability a patient has TB given their protein biomarker levels. The classification by the model was based on log odds, which was calculated as log odds = ln(pr_TB_/pr_non-TB_), where pr is the probability.

For each phase of the study, 80% of the training set samples were used to generate diagnostic models and for cross-validation. The remaining 20% of samples were used as a holdout test set to calculate the model performance estimates prior to application of the models to the blinded verification samples.

## Supplementary Material

Supplemental material
