# Supplementary material for: Discovery and Validation of a Six-Marker Serum Protein Signature for the Diagnosis of Active Pulmonary Tuberculosis
Source: J Clin Microbiol. 2017 Sep 25;55(10):3057–71. doi: 10.1128/JCM.00467-17 (PMC5625392; doi:10.1128/JCM.00467-17)
Supplement: Supplemental material [file supp_55_10_3057__index.html]

Supplemental material 

# Discovery and Validation of a Six-Marker Serum Protein Signature for the Diagnosis of Active Pulmonary Tuberculosis

## Supplemental material

- Supplemental file 1 -

  Fig. S1 (TB biomarker discovery in phase I)

  PDF, 712K
- Supplemental file 2 -

  Fig. S2 (TB biomarker model performance in phase I)

  PDF, 436K
- Supplemental file 3 -

  Fig. S3 (Differences between TB patients and non-TB subjects in the discovery set of the phase II biomarker discovery study with respect to age, BMI, gender, and HIV status)

  PDF, 279K
- Supplemental file 4 -

  Fig. S4 (Stability selection of proteins using a logistic regression model with phase II training samples using protein data alone and augmented by age, gender, site, HIV status, and country)

  PDF, 263K
- Supplemental file 5 -

  Fig. S5 (SDS-PAGE analysis of SOMAmer affinity-capture fractions of kallistatin, C9, gelsolin, and SYWC)

  PDF, 273K
- Supplemental file 6 -

  Fig. S6 (TB host biomarker candidates excluded from models due to inadequate performance)

  PDF, 182K
- Supplemental file 7 -

  Fig. S7 (Confidence score assessment based on the quality of associated metadata used for the “true” classification of all unique samples tested in phase II, TB LogOdds for all phase II samples with high-confidence metadata scores, and ROC curves stratified by HIV and smear status)

  PDF, 234K
- Supplemental file 8 -

  Fig. S8 (Alternative models with two or four TB biomarkers)

  PDF, 440K
- Supplemental file 9 -

  Fig. S9 (Protein-protein interaction network visualized by STRING)

  PDF, 774K
