# Supplementary material for: Discovery and Validation of a Six-Marker Serum Protein Signature for the Diagnosis of Active Pulmonary Tuberculosis
Source: J Clin Microbiol. 2017 Sep 25;55(10):3057–71. doi: 10.1128/JCM.00467-17 (PMC5625392; doi:10.1128/JCM.00467-17)

FIG S1 TB biomarker discovery in Phase 1. A. Demographic and clinical data of samples analyzed by 1129plex SOMAscan. B. Volcano plot of serum proteins differentially expressed in 333 serum samples (TB vs. non-TB). Toward the top are markers with the best KS statistics, and to the extreme right and left are the proteins with the largest median fold-change. C. Stability paths of logistic regression analysis, for protein data alone (upper panel) and protein data augmented by age, gender, site, and country (lower panel). D. Correlation matrices for candidate biomarkers ranked by KS statistics. The color gradient is based on the Spearman rho factor for protein pairs with positive correlation (red), no correlation (white), and negative correlation (blue). E. Correlation matrices for candidate biomarkers stability selection using logistic regression.

A.

Demographic or clinical parameter	Phase I (tested on 1129plex)	
	Training/Test	Blinded Verification
Number of samples tested	450	300
South Africa	151	115
Peru	47	84
Vietnam	252	101
Active TB, culture-confirmed	225 (50%)	150 (50%)
Smear-positive TB	200 (89% of cases)	100 (67% of cases)
Smear-negative TB	25 (11% of cases)	50 (33% of cases)
Included in analysis	419 (93.1%)	270 (90%)
HIV co-infection, n (%)	150 (33%)	120 (40%)
Age, years (range)[a]	36 (17-81), n=445	36 (18-76), n=110
Gender (%male) [a]	65.0%, n=449	60.5%, n=261
Weight, kg (range) [a]	58 (36-92), n=208	Not reported
Height, cm (range) [a]	165 (134-189), n=223	161 (140-175), n=54
BMI, kg m^{-2} (range) [a]	20.9 (14.5-37.7), n=198	Not reported

[a]Reported for a subset of the samples only, as indicated by *n*

B.

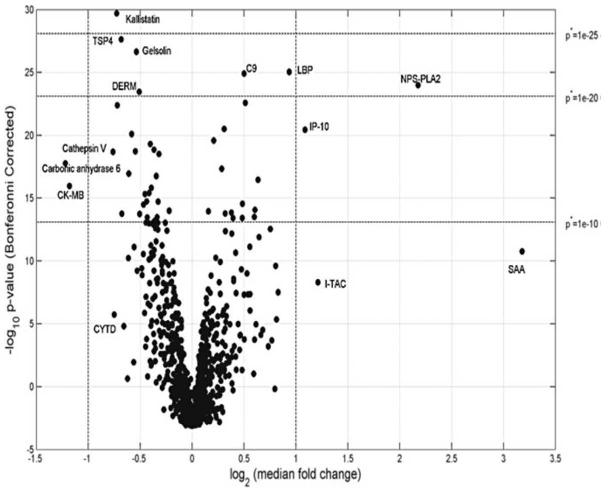

C.

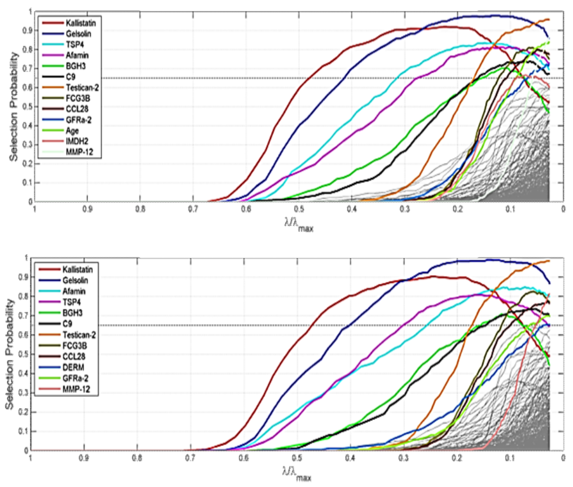

D.

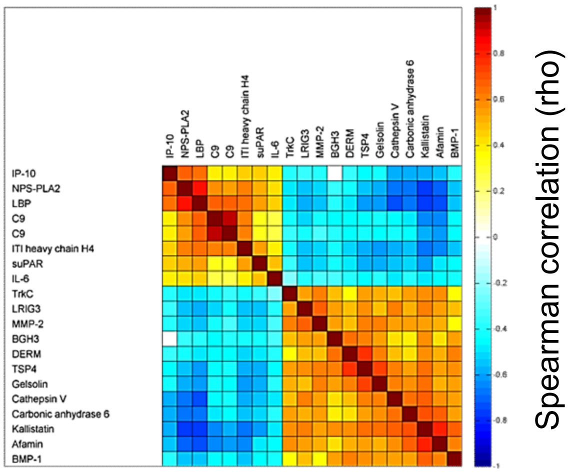

E.

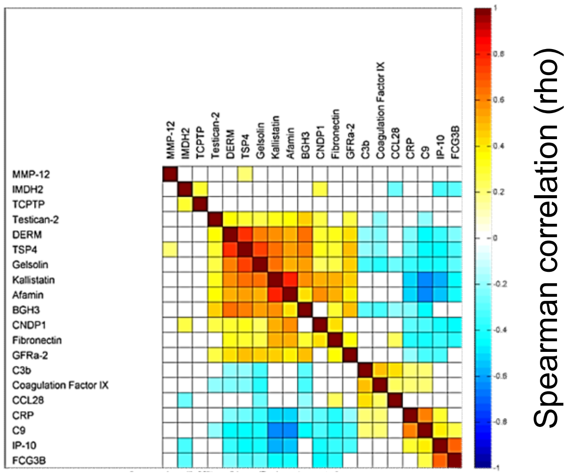

Supplement: Supplemental material [file JCM.00467-17_zjm999095669s1.pdf]
