# Supplementary material for: Discovery and Validation of a Six-Marker Serum Protein Signature for the Diagnosis of Active Pulmonary Tuberculosis
Source: J Clin Microbiol. 2017 Sep 25;55(10):3057–71. doi: 10.1128/JCM.00467-17 (PMC5625392; doi:10.1128/JCM.00467-17)

FIG S2. TB biomarker model performance in Phase I. Combining the most significant univariate features and the most stable features identified by stability selection yielded an optimal model with nine markers: kallistatin, gelsolin, TSP4, afamin, BGH3, C9, testican-2, FCG3B, and DERM. Other markers such as LBP and NPS-PLA2 showed strong negative correlation with the top marker kallistatin and were therefore not included in the model. In contrast, markers such as FCG3B and BGH3 that were identified by stability selection were generally less correlated and therefore able to improve performance of models. A. ROC curve for HR9 (host response 9-marker model) in training, test, and blinded verification set. The colored boxes show the model performance expectations based on the 95% confidence intervals. B. Decision boundary plot based on the LogOdds for TB of 270 verification samples. Red, TB; blue, non-TB; hollow dots, mis-classifications; green, indeterminate due to analytical variability (LogOdds interval [-1.2, +1.3]).

A.

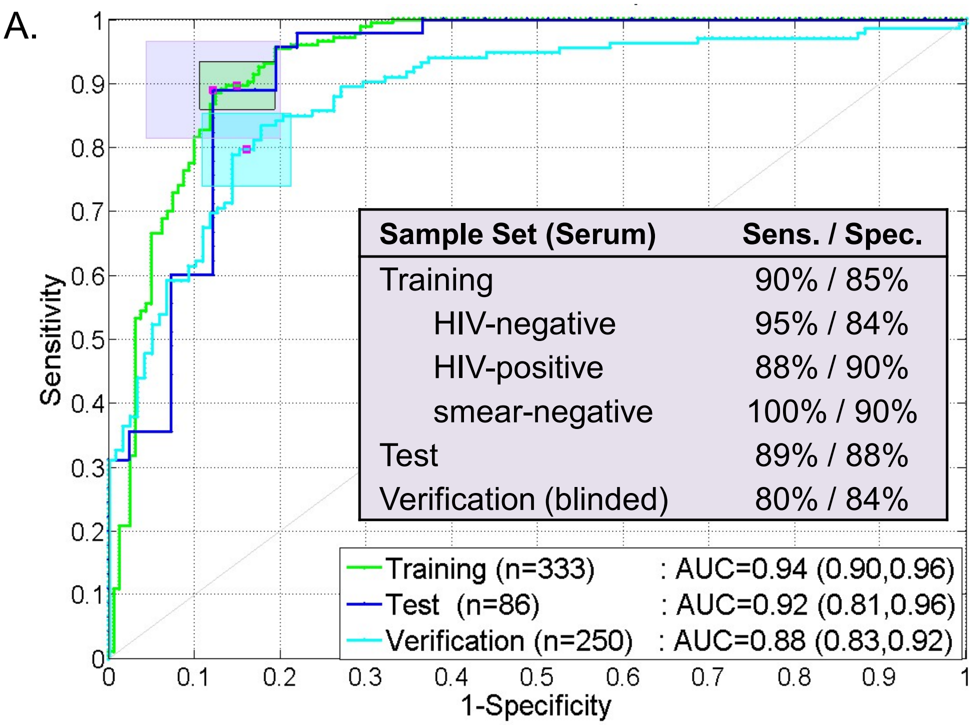

B.

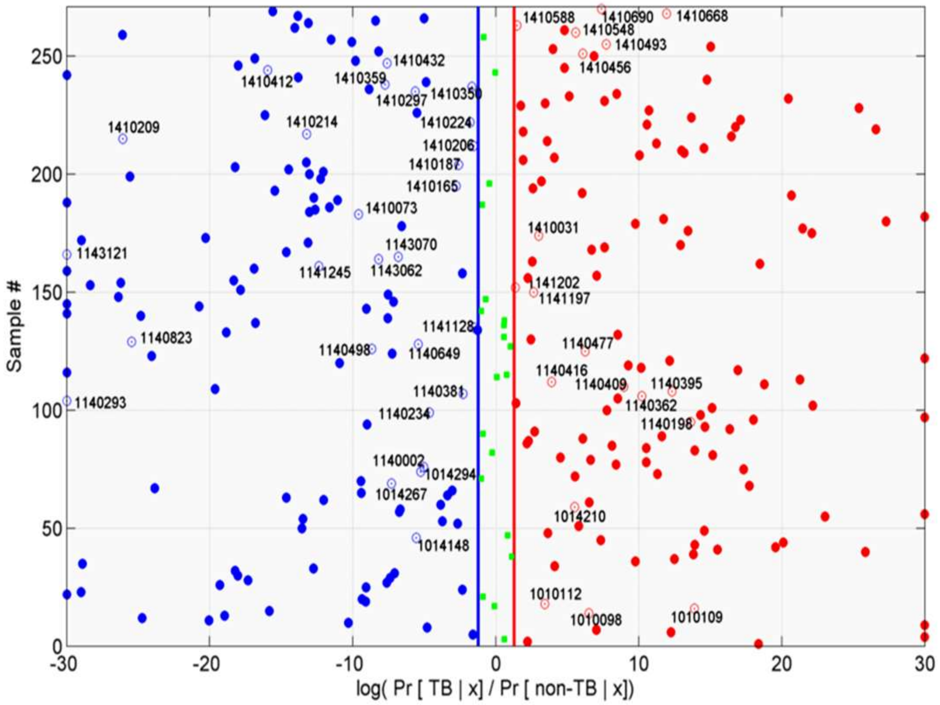

Supplement: Supplemental material [file JCM.00467-17_zjm999095669s2.pdf]
