# Supplementary material for: Discovery and Validation of a Six-Marker Serum Protein Signature for the Diagnosis of Active Pulmonary Tuberculosis
Source: J Clin Microbiol. 2017 Sep 25;55(10):3057–71. doi: 10.1128/JCM.00467-17 (PMC5625392; doi:10.1128/JCM.00467-17)

FIG S3 . Differences between TB patients (red, n=228) and non-TB subjects (blue, n=238) in the discovery (training) set of the Phase II biomarker discovery study with respect to age (A), BMI (B), gender (C), and HIV status (D).

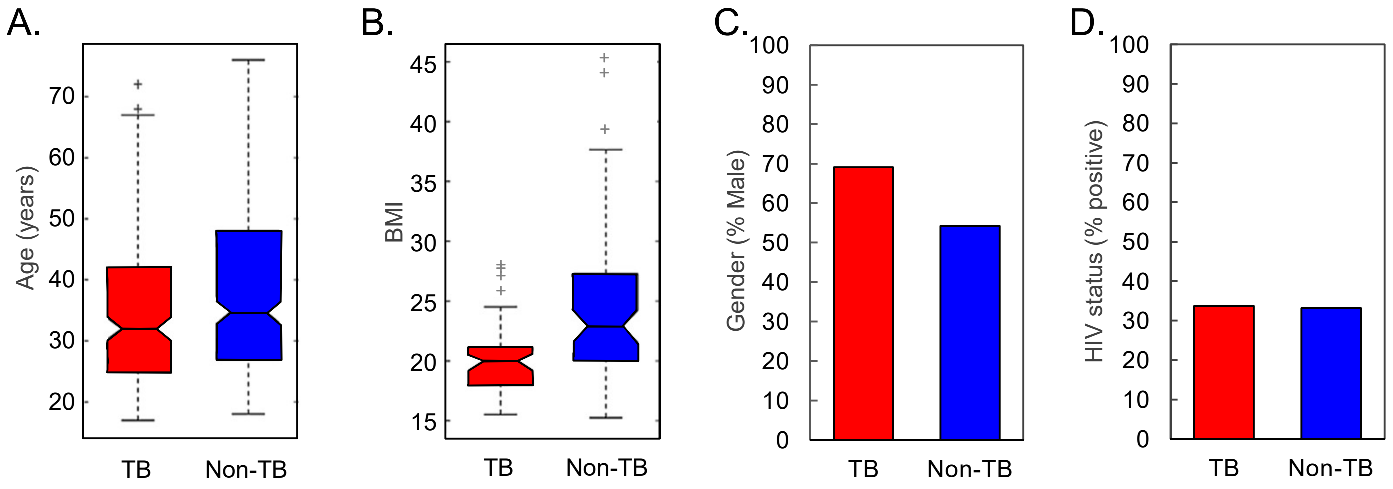

Supplement: Supplemental material [file JCM.00467-17_zjm999095669s3.pdf]
