# Supplementary material for: Discovery and Validation of a Six-Marker Serum Protein Signature for the Diagnosis of Active Pulmonary Tuberculosis
Source: J Clin Microbiol. 2017 Sep 25;55(10):3057–71. doi: 10.1128/JCM.00467-17 (PMC5625392; doi:10.1128/JCM.00467-17)

FIG S4 Stability selection of proteins using a logistic regression model with Phase II training samples (n=228 TB vs. n=238 non-TB, stability paths Pw=0.50, weakness=0.90) using protein data alone (A) and augmented by age, gender, site, HIV status, and country (B).

A.

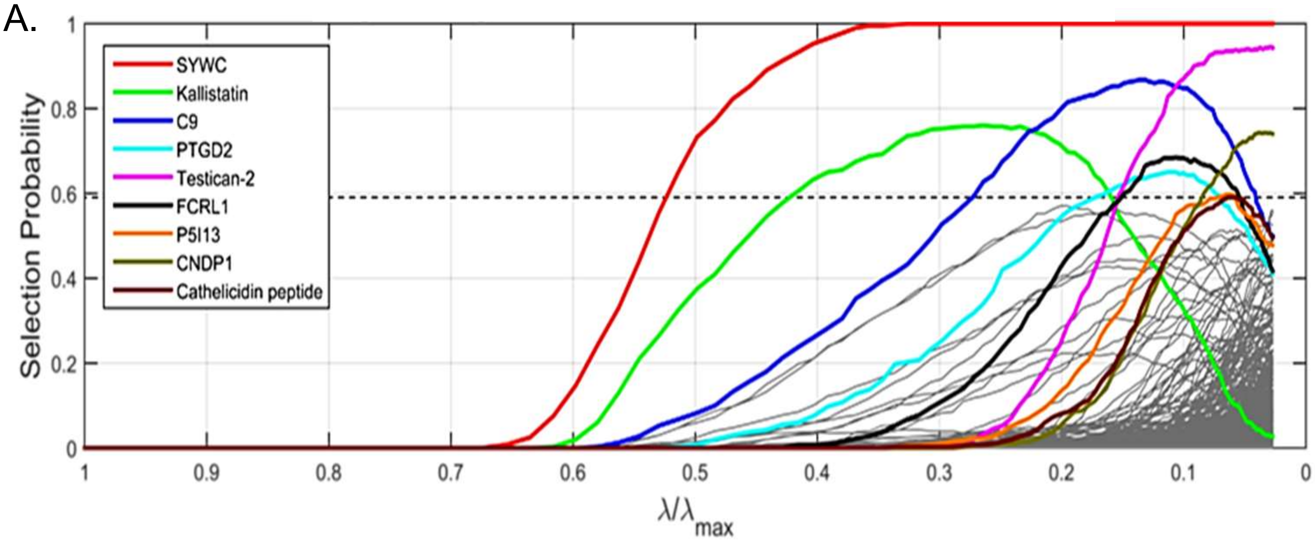

B.

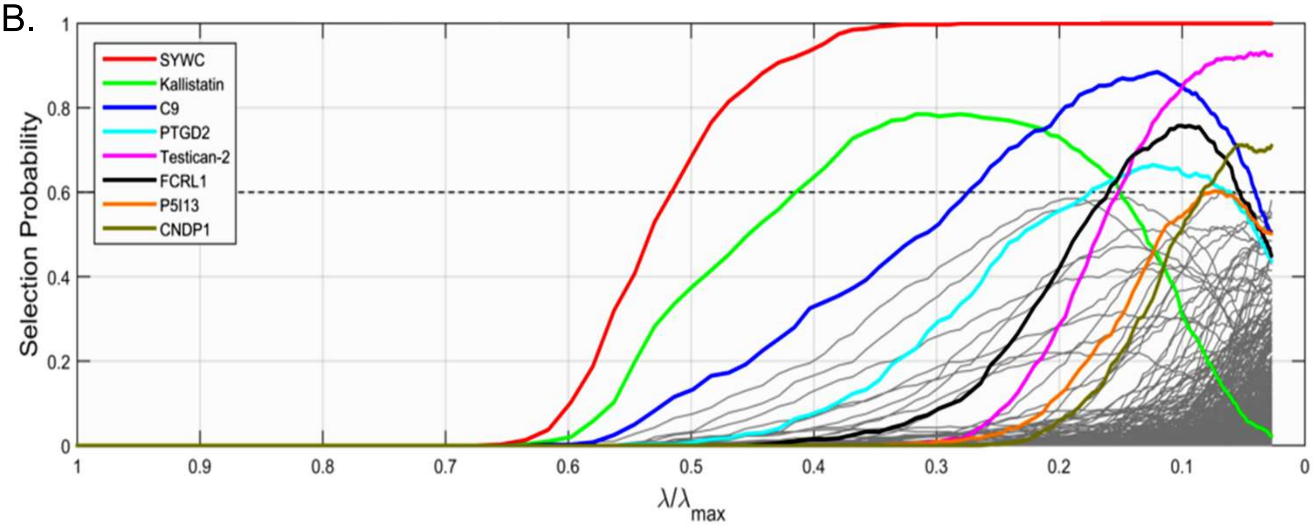

Supplement: Supplemental material [file JCM.00467-17_zjm999095669s4.pdf]
