# Supplementary material for: Discovery and Validation of a Six-Marker Serum Protein Signature for the Diagnosis of Active Pulmonary Tuberculosis
Source: J Clin Microbiol. 2017 Sep 25;55(10):3057–71. doi: 10.1128/JCM.00467-17 (PMC5625392; doi:10.1128/JCM.00467-17)

FIG S5 SDS-PAGE analysis of SOMAmer affinity-capture (pull-down) fractions of kallistatin (lanes 1-4), C9 (lanes 5-8), gelsolin (lanes 9-12), and SYWC (lanes 13-16). The four lanes per target show recombinant protein, affinity-capture of recombinant protein from buffer, affinity-capture of recombinant protein from 40% human serum, and affinity-capture of native (endogenous) protein from 40% human serum. Kallistatin, C9, and gelsolin were pulled down from 50 µl serum and SYWC was pulled-down from 400 µl. Proteins captured by bead-immobilized SOMAmers were tagged with NHS-Also shown is the non-specific background when using beads alone for pull-downs (lanes 17-20). M, molecular weight marker. A. Cy5 filter to visualize Alexa Fluor® Dye647-labeled proteins.
B. Cy3-filter, to visualize Cy3-labeled SOMAmers used for pull-down. Since specific SOMAmer-target interactions occur with 1:1 stoichiometry, protein and SOMAmer show comparable signal intensities.

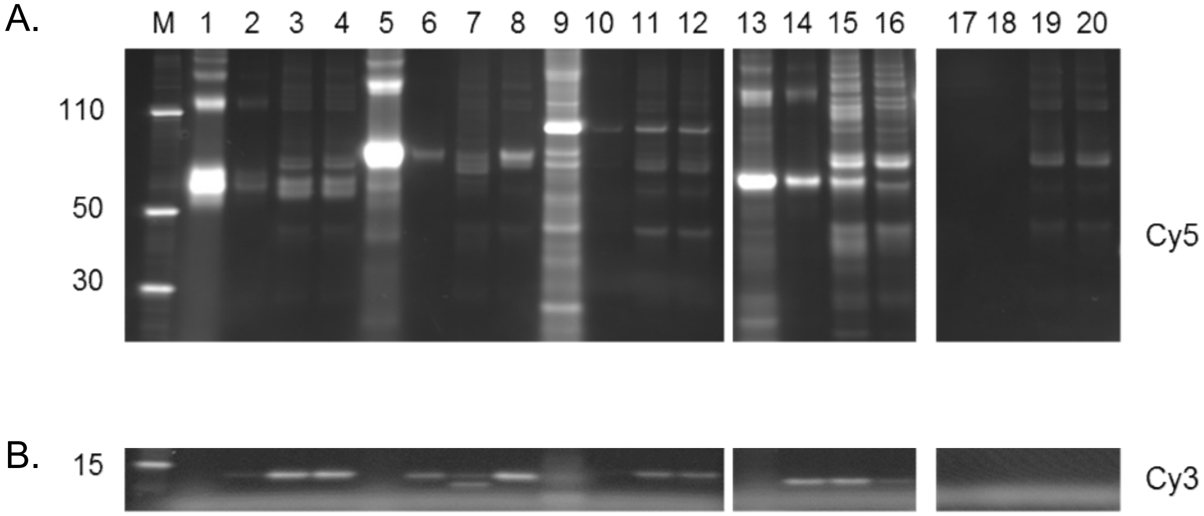

Supplement: Supplemental material [file JCM.00467-17_zjm999095669s5.pdf]
