# Supplementary material for: Discovery and Validation of a Six-Marker Serum Protein Signature for the Diagnosis of Active Pulmonary Tuberculosis
Source: J Clin Microbiol. 2017 Sep 25;55(10):3057–71. doi: 10.1128/JCM.00467-17 (PMC5625392; doi:10.1128/JCM.00467-17)

FIG S6 TB host biomarker candidates excluded from models due to inadequate performance. A. CCL28 as an examples of a TB biomarker candidate with site (country)-dependent signals. B. sCD163 as an example of TB biomarker candidates affected by HIV status. Boxes represent 75th-25th percentiles, whiskers represent minimum and maximum range of all data points, and the horizontal bars represents the median values.

A.

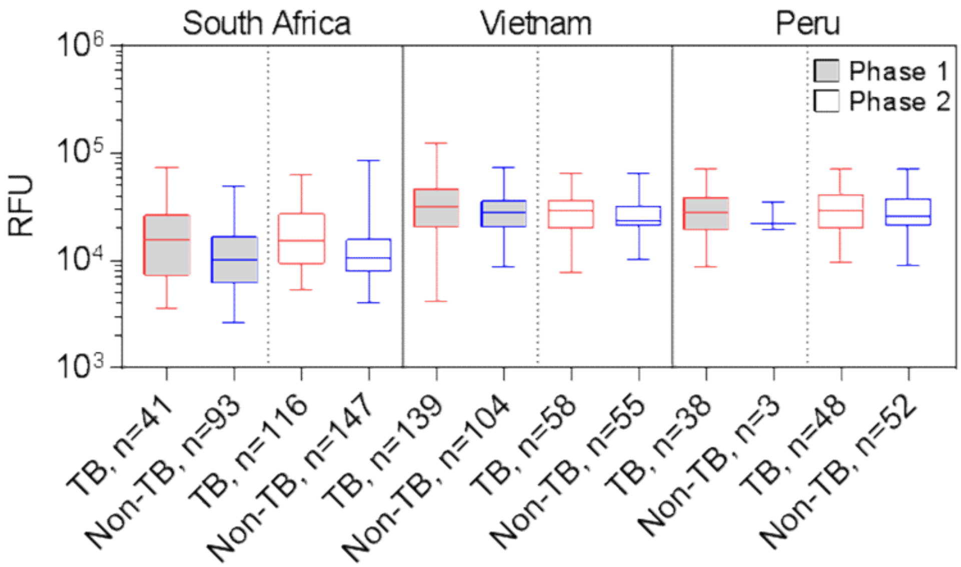

B.

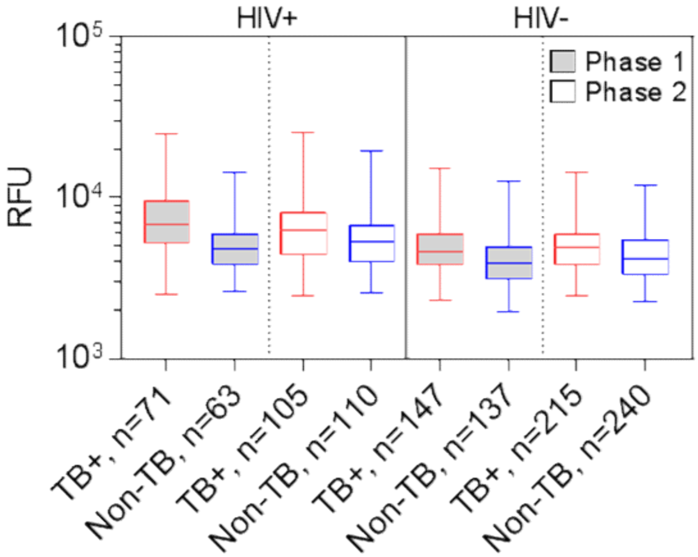

Supplement: Supplemental material [file JCM.00467-17_zjm999095669s6.pdf]
