# Supplementary material for: Discovery and Validation of a Six-Marker Serum Protein Signature for the Diagnosis of Active Pulmonary Tuberculosis
Source: J Clin Microbiol. 2017 Sep 25;55(10):3057–71. doi: 10.1128/JCM.00467-17 (PMC5625392; doi:10.1128/JCM.00467-17)

FIG S7 A. Confidence score assessment based on the quality of associated meta data used for the ‘true’ classification (non-TB or TB) of all unique samples tested in Phase II. Abbreviations: SP, sputum culture; FU, follow-up; SC, solid culture; LC, liquid culture; Xp, Gene Xpert data; QFT, quantiferon test (interferon-γ release assay); sm, smear. B.TB LogOdds for all Phase II samples (n=616) with high-confidence metadata scores. True non-TB (blue) and true TB (red) samples were ordered by their HR6 LogOdds score. The dotted line indicated the decision boundary (LogOdds = 0) for classification by the HR6 model. False-positives and false-negatives are indicated by the blue and red brackets, respectively. C. All unique Phase II samples with high-confidence metadata (n=569) were used to generate ROC curves stratified by HIV and smear status. D. Subgroup analysis for a more restrictive subset of only those samples that had not been used in training of the model, stratified by HIV and smear status.

A.

Score	Non-TB (n=323)	N (%)	TB (n=295)	N (%)
1	≥2 SPs neg and FU cultures neg	171 (52.9%)	≥2 SPs pos on both SC and LC, or SC or LC pos on 2 SPs and Xp pos	200 (67.8%)
0.75	≥2 SPs neg, no FU cultures, but improved/recovered, or Only 1 SP neg, but Xp or QFT neg	136 (42.1%)	2 SPs pos, but 1-2 cultures neg, or 2 SC pos or 2 LC pos, and sm or Xp pos	62 (21.0%)
0.5	≥2 SPs neg, but no FU or same/worse symptoms at FU, or only 1 SP neg and extensive lack of meta data	7 (2.2%)	Only 1 SP pos, but sm/Xp pos or FU, or 1-2 positive cultures and Xp low quant, passed away	18 (6.1%)
0.25	Insufficient meta data for neg culture status	0 (0%)	Only 1 SP, no FU and 2 cultures contam/lost, or no culture data but 2 sm pos	11 (3.7%)
0	No meta data for neg culture status	9 (2.8%)	Only 1 SP pos w/o sm/Xp/FU, or No meta data for pos culture status	4 (1.4%)

B.

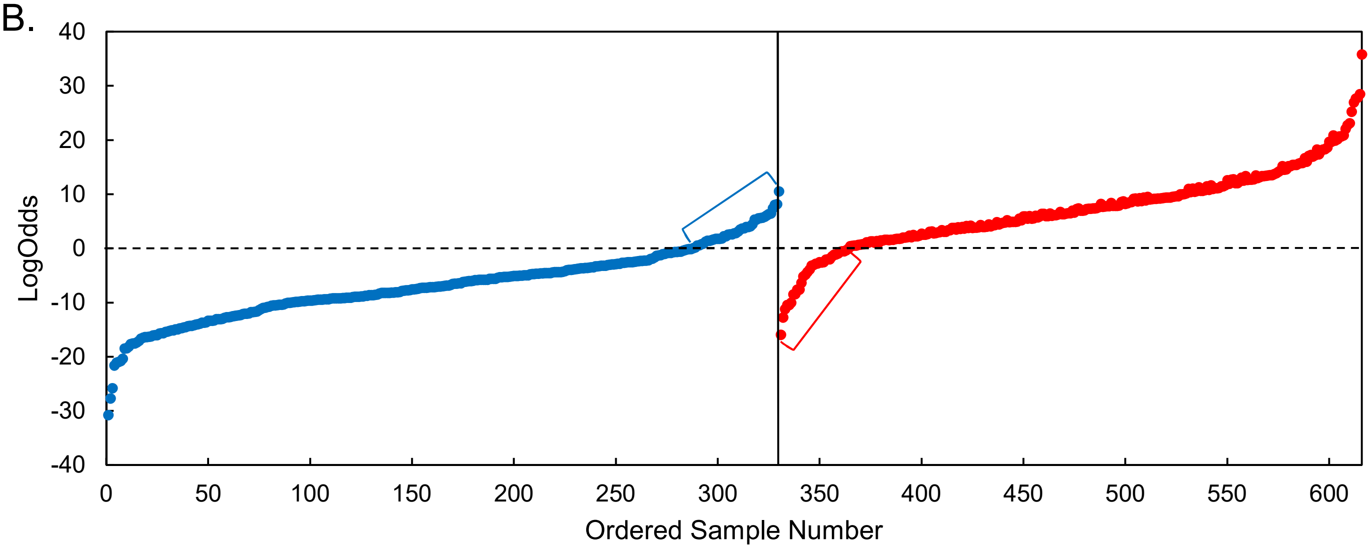

C.

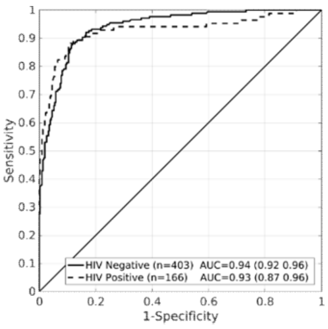

D.

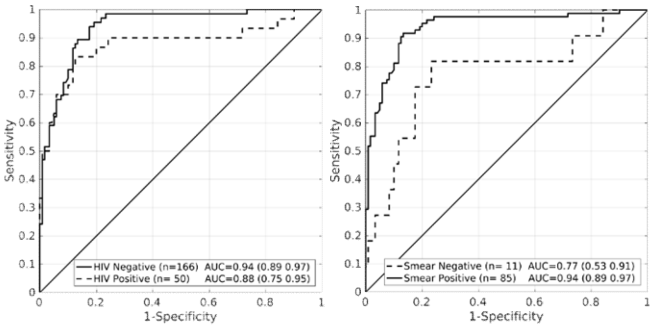

Supplement: Supplemental material [file JCM.00467-17_zjm999095669s7.pdf]
