# Supplementary material for: Discovery and Validation of a Six-Marker Serum Protein Signature for the Diagnosis of Active Pulmonary Tuberculosis
Source: J Clin Microbiol. 2017 Sep 25;55(10):3057–71. doi: 10.1128/JCM.00467-17 (PMC5625392; doi:10.1128/JCM.00467-17)

FIG S8. Alternative models with two or four TB biomarkers. A. Bivariate scatter plot for a two-protein relevance vector machine model using kallistatin and SYWC. Solid symbols, TB (red) and non-TB (blue); hollow symbols, misclassified samples. The points emphasized by circles served to anchor the decision boundary line (black). B. Signal distributions of four proteins (SAA, NPS-PLA2, IP-10, CA-6) that had the largest median fold-change between TB and non-TB. Boxes represent 75th-25th percentiles, whiskers represent minimum and maximum range of all data points, and the bars represent the median values. C. Cdf of a 4-marker model (HR4) compared to the 6-marker model HR6, separating TB from non-TB. D. ROC curves for the HR4 model in training, test, and verification sample set. The colored boxes represent performance estimates based on the associated confidence intervals.

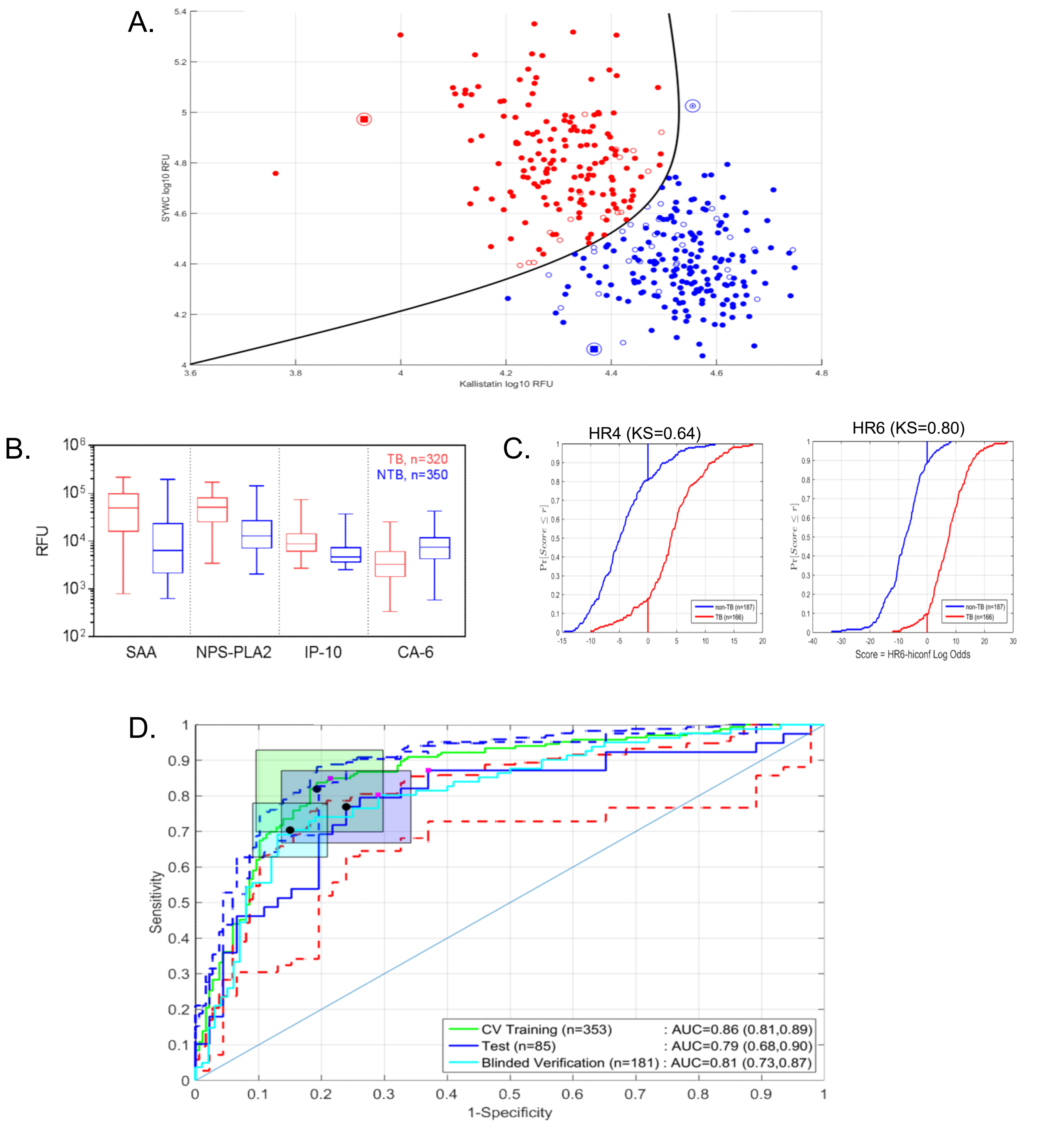

Supplement: Supplemental material [file JCM.00467-17_zjm999095669s8.pdf]
