# Supplementary material for: Discovery and Validation of a Six-Marker Serum Protein Signature for the Diagnosis of Active Pulmonary Tuberculosis
Source: J Clin Microbiol. 2017 Sep 25;55(10):3057–71. doi: 10.1128/JCM.00467-17 (PMC5625392; doi:10.1128/JCM.00467-17)

FIG S9 Protein-protein interaction network visualized by STRING (Search Tool for the Retrieval of Interacting Genes/Proteins). The edges (lines) represent a minimum interaction confidence score of 0.4. The colors of the edges indicate data from the following: yellow, text mining; green; gene neighborhood, red; gene fusions, blue; gene co-occurrence, black; co-expression, light blue; protein homology

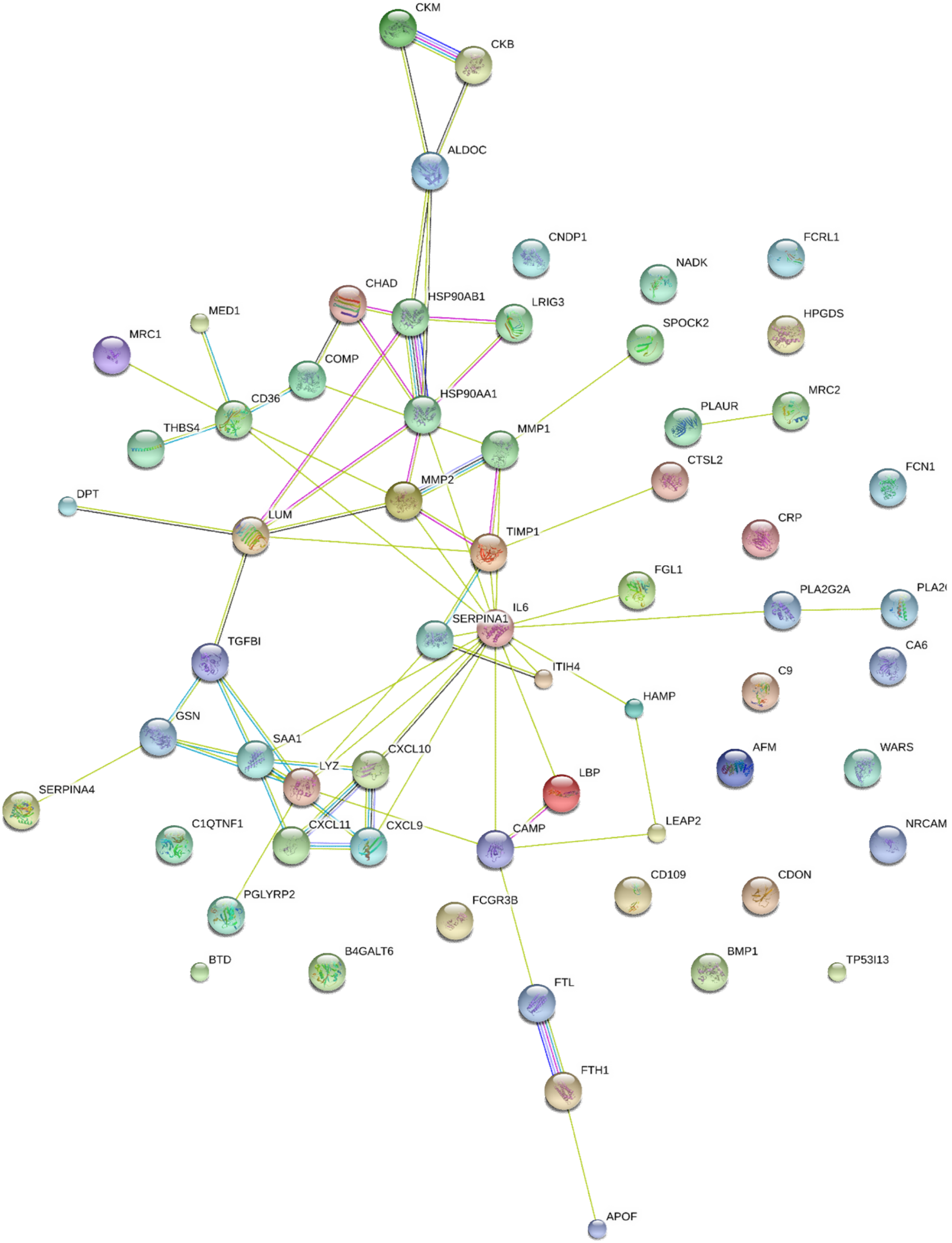

Supplement: Supplemental material [file JCM.00467-17_zjm999095669s9.pdf]
